# Unraveling the therapeutic potential of *Elaeagnus angustifolia* extract on triple-negative breast cancer (TNBC): An investigation using zebrafish model

**DOI:** 10.1371/journal.pone.0344247

**Published:** 2026-03-06

**Authors:** Haya Abuhijleh, Zain Zakria, Hiba Bawadi, Ayat Hammad, Ala-Eddin Al Moustafa, Abdullah Shaito, Maha Al-Asmakh

**Affiliations:** 1 Department of Biomedical Sciences, College of Health Sciences, QU Health, Qatar University, Doha, Qatar; 2 Department of Nutrition Sciences, College of Health Sciences, QU Health, Qatar University, Doha, Qatar; 3 Vice President for Medical and Health Sciences Office, QU Health, Qatar University, Doha, Qatar; 4 Oncology Department, Faculty of Medicine, McGill University, Montreal, QC, Canada; 5 Biomedical Research Center, Qatar University, Doha, Qatar; Cairo University, Faculty of Science, EGYPT

## Abstract

Breast cancer is a widespread and aggressive disease, with 2.3 million new cases globally in 2022. Metastasis, the spread of cancer cells to distant organs, remains a leading cause of breast cancer-related mortality. Current treatment options, particularly traditional chemotherapeutic drugs, are often associated with severe side effects, emphasizing the urgent need for safer and more effective therapeutic alternatives. Triple-negative breast cancer (TNBC) represents one of the most aggressive breast cancer subtypes, characterized by the absence of estrogen receptors (ER), receptors (PR), and HER2 expression. The human TNBC cell line MDA-MB-231, was selected in this study due to its aggressive, metastatic phenotype and its well-established use in zebrafish xenograft models. This makes it a highly relevant platform for preliminary *in vivo* evaluation of novel plant-derived compounds, particularly those targeting hard-to-treat breast cancer subtypes such as TNBC. *Elaeagnus angustifolia* (EA), commonly known as Russian olive, has attracted interest for its antimicrobial, anti-inflammatory, and antioxidant properties. However, its potential anticancer activity, especially against TNBC, remains relatively unexplored. This research investigated the efficacy of EA extract against MDA-MB-231 TNBC cells using a wild-type AB zebrafish model. A key objective was to evaluate the toxicological profile of EA across multiple physiological parameters in zebrafish, including developmental, cardiovascular, neuromuscular, and hepatic functions. The study identified safe, non-toxic concentrations of EA extract (0.5 mg/mL and 0.75 mg/mL). Moreover, treatment with EA in zebrafish xenografts led to a dose-dependent reduction in fluorescence intensity of injected TNBC cells, suggesting suppression of tumor cell proliferation and survival.. These findings suggest that EA warrant further investigation as a potential anticancer agent for TNBC. The observed safety profile and preliminary anti-tumor effects in zebrafish provide a foundation for future mechanistic and mammalian studies.

## Background

Breast cancer remains the most commonly diagnosed cancer among women, with an estimated 2.3 million new cases and 670,000 deaths globally in 2022. It was the most common cancer in women in 157 of 185 countries, and lifetime risk varies strikingly by development level – from 1 in 12 women in very high human development index countries to 1 in 27 in low human developmental index countries [1]. Over recent decades, classification paradigms have shifted from histological subtypes (e.g., ductal, inflammatory, or invasive types) [2] to intrinsic molecular classifications based on the presence or absence of three key biomarkers: estrogen receptor (ER), progesterone receptor (PR), and human epidermal growth factor receptor 2 (HER2) protein [3,4]. This molecular classification allows for categorization into ER-positive (ER+), PR -positive (PR+), HER2-positive (HER2+), or triple-negative (TNBC) subtypes. Approximately 70% of breast cancer falls under the ER/PR+ category, while about 20% is HER2+ [5,6]. Tumors lacking all three receptors are classified as TNBC [7], and they account for 10–15% of all breast cancers and represents one of the most aggressive subtypes [8]. Unlike hormone receptor-positive or HER2-positive cancers, TNBC lacks a single dominant therapeutic target, which contributes to poor prognosis and limited treatment options. Patients often experience early relapse and high rates of metastasis despite chemotherapy [9,10].

Breast cancer treatment options vary depending on molecular subtype [6,11]. ER/PR-positive tumors generally respond to endocrine therapy such as tamoxifen [12]. HER2-positive breast cancer benefits from targeted therapy with trastuzumab (Herceptin) [13]. However, TNBC, which lacks ER, PR, and HER2 expression, remains a major therapeutic challenge [14,15]. Without molecular targets, chemotherapy remains the mainstay of TNBC management, yet 30–50% of patients experience metastasis even after early-stage treatment [16,17]. This underscores the urgent need for possible therapeutic strategies capable of targeting the aggressive and metastatic nature of TNBC [18–20].

TNBC progression is strongly associated with epithelial–mesenchymal transition, a process that enhances invasiveness and drug resistance. In addition, dysregulation of p53 -a pathway frequently altered in TNBC – contributes to uncontrolled proliferation and impaired apoptosis [21]. TNBC tumors also exhibit heightened angiogenesis and reliance on Ras/ERK signaling cascades, further driving aggressive growth and metastatic spread [21]. These molecular hallmarks underscore the urgent need for therapies that can simultaneously target multiple pathways.

Metastasis, the dissemination of cancer cells to distant organs, is the primary cause of mortality in breast cancer patients [22,23]. While metastatic breast cancer is classified as stage IV, current treatment strategies largely mirror those for earlier stages, with limited success in preventing disease progression [19]. Preventing or delaying metastasis remains a key research priority, and neoadjuvant therapies are being explored as a preventive strategy to eliminate disseminated cancer cells before they establish secondary tumors [24,25].

The development of effective anti-cancer agents, particularly those targeting metastasis, requires robust model systems that replicate tumor invasion, migration, and proliferation. *In vitro* models, while valuable, must be complemented by *in vivo* systems that allow real-time observation of tumor behavior within a living organism [24–26]. Zebrafish (Danio rerio) xenograft models have emerged as a powerful preclinical platform for such studies, offering transparency for live imaging, rapid assay timelines, and high genetic and physiological similarity to humans.

In this study, we selected the MDA-MB-231 cell line, a well-characterized human triple-negative breast cancer cell line, as our tumor model. Originally derived in 1973 from the pleural effusion of a patient with metastatic adenocarcinoma, MDA-MB-231 cells lack ER, PR, and HER2 expression and exhibit a mesenchymal-like morphology, high migratory capacity, and an invasive phenotype [27]. They are also enriched for cancer stem-like features (CD44 ⁺ /CD24^low) and display downregulation of tight junction proteins such as claudin-3 and claudin-4, which contribute to their metastatic potential [28,29].

Importantly, MDA-MB-231 is the most frequently used TNBC line in zebrafish xenograft studies (58% of reported cases) [30,31], where it reliably establishes proliferative cancer cell clusters, disseminates to distant sites (including bone and lung), and allows real-time monitoring of tumor proliferation, migration, and apoptosis under treatment conditions [32,33]. Zebrafish-selected MDA-MB-231 subpopulations have been shown to adopt even more invasive phenotypes via epithelial–mesenchymal transition, further validating their relevance for metastasis research [34]. These characteristics make MDA-MB-231 an optimal choice for preliminary *in vivo* screening of novel plant-derived compounds targeting aggressive and treatment-resistant breast cancer subtypes such as TNBC.

Natural products have long been recognized as a rich source of structurally diverse and biologically active molecules for cancer drug discovery. *Elaeagnus angustifolia* (EA), commonly known as Russian olive, is native to the Middle East and has been traditionally used for its antifungal, antibacterial, anti-inflammatory, and antioxidant properties [35–40]. Flavonoids present in EA have been reported to modulate the tumor suppressor p53, induce G2/M phase cell cycle arrest, suppress Ras protein expression, and regulate heat shock proteins in various malignancies [41]. Although the anticancer potential of EA has been demonstrated in select cancer models, its activity against breast cancer and the underlying molecular mechanisms remain insufficiently explored [42,43]. Notably, recent studies have shown that EA can induce apoptosis and inhibit epithelial–mesenchymal transition through activation of the JNK signaling pathway in HER2-positive breast cancer cells *in vitro* [43]. In addition, hydroalcoholic extracts of EA have been found to significantly suppress angiogenesis, a key hallmark of malignancy [44]. Further evidence from human head and neck cancer cell models indicates that EA promotes cell differentiation, reduces invasive capacity, and restores the E-cadherin/catenin complex via activation of the ERK1/ERK2 pathway [37]. Despite these promising findings, its effects against TNBC remain largely unexplored.

Prior studies on EA therefore provide mechanistic evidence highly relevant to TNBC biology. EA extracts have been shown to induce apoptosis via p53 activation, suppress epithelial-mesenchymal transition, and inhibit angiogenesis -processes that directly contribute to TNBC aggressiveness. This overlap between EA’s mechanisms and TNBC’s vulnerabilities provides a strong rationale for investigating EA in this subtype. Based on these mechanistic overlaps, we hypothesized that EA extract could exert therapeutic effects against TNBC by modulating epithelial-mesenchymal transition, apoptosis, and angiogenesis. This study therefore aimed to evaluate the safety and anti-tumor efficacy of EA aqueous flower extract in a zebrafish xenograft model, providing preliminary in vivo evidence for its potential as a novel therapeutic agent.

## Research design and methodology

All animal experiments were conducted in accordance with national and international regulations governing zebrafish research [45]. The study protocol was approved by Qatar University’s Institutional Animal Care and Use Committee (QU-IACUC-006/2023) and adhered to the university’s established animal protocol standards. In addition, the research complied with the Policy on Zebrafish Research issued by the Department of Research, Ministry of Public Health, Qatar (2017). The experimental design also receiveƒd approval from Qatar University’s Institutional Biosafety Committee (IBC) under document QU-IBC-2022/014.

Zebrafish welfare was prioritized throughout their life cycle. Adult zebrafish breeding was carried out in line with ethical guidelines, ensuring appropriate care. Embryos were maintained in carefully controlled conditions to promote optimal development, and humane euthanasia was performed using tricaine solutions to minimize suffering and distress. Death was confirmed by the absence of both heartbeat and movement, in accordance with established ethical procedures.

To comply with IBC and IACUC requirements at Qatar University, all researchers involved in the study completed mandatory training through the CITI Program. Training modules included Working with Animals in Biomedical Research, Working with Fish in Research Settings, and Working with Zebrafish (Danio rerio) in Research Settings. This preparation ensured adherence to ethical standards and responsible conduct of animal research.

Humane endpoints were applied for all zebrafish embryos. Euthanasia criteria included signs of severe suffering or distress, such as marked behavioral changes, reduced mobility, abnormal posture, and other clinical indicators of poor prognosis and diminished quality of life. No animals were euthanized before meeting these humane endpoint criteria.

For euthanasia, zebrafish larvae were exposed to an overdose of MS-222 far exceeding anesthesia doses. They were maintained in a tricaine solution at a concentration of 300 mg/L for at least 30 minutes after all visible movement had ceased, as recommended by Matthews and Varga (2012) [46]. This extended exposure ensured complete cessation of brain activity and thorough completion of the euthanasia process.

### *Elaeagnus angustifolia* extract preparation

Dried *EA* plant material was collected from Montreal, Quebec (Latitude: 45.527427, Longitude: –73.687523; Saint Laurent Park, 845 Poirier Street, Saint-Laurent, Montreal, QC H4L 1G4, Canada). Flowers were harvested at approximately 6:00 PM during the second week of June, shade-dried, and stored in a dark container at room temperature (~23 °C) until use.

Extract preparation followed previously established protocols [47,48] with minor modifications. Briefly, 750 mg of the dried plant material was weighed using a calibrated balance and added to 50 mL of Embryo Medium (E3M) in a flask. The mixture was boiled for 20 minutes at 100 °C on a hot plate with continuous magnetic stirring. To minimize evaporation, the flask was covered with aluminum foil during boiling. The resulting solution was centrifuged at 2,500 rpm for 3 minutes and filtered through a standard inverted flask fitted with filter paper. Fresh dilutions of the extract were prepared immediately prior to each experiment.

### Zebrafish maintenance and breeding

#### Housing and husbandry.

Adult zebrafish (*Danio rerio*, AB strain) were housed in a recirculating stand-alone aquarium system at the Qatar University Biomedical Research Center. The facility was maintained at a controlled ambient temperature of 26 °C, with water temperature stabilized at approximately 28 °C. A photoperiod of 14 hours light (07:30–21:30) followed by 10 hours dark (21:30–07:30) was implemented to replicate natural day/night cycles [49].

#### Breeding and embryo collection.

To obtain high-quality embryos, zebrafish breeding was carried out following established laboratory protocols [24]. On the evening prior to breeding, adult zebrafish were separated by sex using a mesh divider placed within the breeding tank. At the onset of the light cycle the next morning, the divider was removed, permitting mating to occur in shallow water for approximately 20 minutes. Fertilized eggs were then collected and gently rinsed with Embryo Medium (E3M) prepared according to the method described by Westerfield [49].

#### Experimental groups and incubation.

Collected embryos were allocated into experimental groups, with 30 embryos per group (n = 30). Each treatment category was tested in triplicate and included: a negative control (E3M only), a positive control (20 μg/mL zinc oxide), and graded concentrations of *EA* extract selected based on prior optimization trials to establish a non-lethal dosage range. Embryos were incubated at a constant temperature of 28 °C, and dead embryos were removed daily with minimal disturbance to the E3M medium. Only wild-type AB zebrafish embryos in less than 5 days post-fertilization, in accordance with QU-IBC approval (QU-IBC-2022/014), were used. Observations were conducted between 24 and 96 hours post fertilization (hpf).

#### Maximum tolerated concentration (MTC) and no observed adverse effect level (NOAEL) in zebrafish larvae.

In toxicology studies involving aquatic organisms such as zebrafish larvae, the Maximum Tolerated Concentration (MTC) and the No Observed Adverse Effect Level (NOAEL) are key parameters for defining safe exposure limits [50–52]. The MTC represents the highest concentration of EA extract that does not significantly reduce survival compared with the negative control group (E3M) over a defined exposure period, typically 96 hours [53]. This value establishes the upper threshold beyond which lethality occurs. The NOAEL is defined as the highest concentration at which no statistically significant increase in adverse effects—such as visible malformations including curved body axis, yolk sac edema, or pericardial edema—is observed compared with the negative control group [54]. Together, these parameters guide the selection of concentrations that are both safe and biologically relevant for subsequent experimental work.

For this study, the optimal concentration used in downstream experiments was deliberately chosen to be lower than both the MTC and NOAEL. This ensured that subsequent investigations focused on the biological effects of the extract without inducing lethality or developmental abnormalities.

To determine the MTC and NOAEL, zebrafish embryos were first exposed to a range of EA extract concentrations at 6 hpf, while the negative control group received E3M only. Each treatment group contained 10 embryos, with three independent replicates. Observations were conducted every 24 hours over the subsequent 96-hour exposure period, aligned with the developmental stages required for the downstream experiments, and concluded at 5 days post-fertilization (120 hpf). This schedule ensured continuous monitoring across four consecutive days. During each observation, survival and the presence of morphological abnormalities (e.g., curved body axis, yolk sac edema, pericardial edema) were assessed using a stereomicroscope. Based on these findings, the concentration that produced no overt developmental defects or larval toxicity was selected for subsequent experiments. Additionally, conventional endpoints such as survival rate, hatching rate, and assessments of cardiac and hepatic toxicity were evaluated to further characterize any potential toxic effects.

#### Toxicity assays for treatments.

A series of conventional toxicity assays was conducted on zebrafish embryos and larvae to assess the potential adverse effects of EA treatments on early development.

#### Developmental toxicity: Survival rate and hatching rate analyses.

The effects of EA treatments on embryo viability were evaluated at key developmental stages. Survival rates were recorded at 24, 48, and 72 hpf using an Axiovert 40 CFL inverted confocal microscope (Carl Zeiss AG). Hatching rates were determined at 48 and 72 hpf by calculating the percentage of embryos that successfully emerged from their chorions within each treatment group.

#### Cardiotoxicity assessment: Live imaging of zebrafish.

To evaluate the potential effects of EA treatments on cardiovascular development and function, a detailed cardiotoxicity assessment was performed on treated embryos and negative controls (E3M) at 3 days post-fertilization (dpf). High-speed time-lapse recordings were obtained using a SteREO Discovery V8 light microscope (Carl Zeiss AG) equipped with a Hamamatsu Orca Flash camera (Hamamatsu Photonics UK Limited) and HCImage software version 2.0.4 (Hamamatsu Photonics UK Limited).

Recordings were examined for gross cardiac morphology, with particular attention to the presence of edema and major structural abnormalities such as looping defects. In addition, the time-lapse sequences were analyzed to assess cardiac function. Red blood cell (RBC) movement within major vessels and ventricular contractions were tracked using an in-house algorithm implemented in Viewpoint software (version 3.4.4, Lyon, France). From these data, blood velocity within the cardiovascular system was calculated, and frictional shear stress was derived in accordance with previously published zebrafish heart function analysis protocols [55,56].

#### Neuromuscular toxicity: Behavioral and locomotion assay.

A locomotor activity assay was conducted at 96 hpf to assess the potential effects of EA treatments on zebrafish embryo movement patterns. Individual embryos were placed in the wells of a 96-well plate positioned within the Viewpoint ZebraLab chamber (Noldus Information Technology, Wageningen, The Netherlands), a system designed for automated digital monitoring of embryonic movements. Locomotor activity was recorded for 60 minutes using the Viewpoint MicroZebraLab tracking software (version 3.4.4, Lyon, France).

Recordings were performed under alternating light and dark cycles to replicate natural environmental conditions experienced by zebrafish. The software quantified multiple movement parameters for each treatment group, including total distance traveled, average swimming speed, and time spent in active motion [57]. This analysis enabled the identification of treatment-induced changes in locomotor activity, providing insight into potential neuromuscular toxicity.

#### Oil red O staining (hepatotoxicity).

Hepatotoxicity was assessed using Oil Red O (ORO) staining, initiated by fixing zebrafish embryos at 5 days post-fertilization (dpf) [58]. Embryos were anesthetized with a few drops of 4 mg/mL tricaine (pH 6.9) added to the water, followed by gentle swirling of the Petri dish to ensure even anesthetic distribution. The larvae were then transferred to microcentrifuge tubes, washed twice with cold PBS, and immersed in 1 mL of 4% paraformaldehyde in PBS. Fixation was carried out overnight at 4 °C.

On the following day, a fresh 0.25% ORO working solution was prepared by stirring the ORO stock solution on a magnetic stirrer for 5–10 minutes, mixing an aliquot with 1 mL of 10% isopropanol (in Milli-Q water), and allowing it to stand at room temperature for 20 minutes before filtration through a 0.2 μm syringe filter. Fixed larvae were washed for 1 hour in 60% isopropanol on an orbital shaker, immersed in the 0.25% ORO working solution for 1 hour and 15 minutes, and subsequently washed with 60% isopropanol followed by 0.1% Tween in PBS.

To prevent drying, 1–2 mL of PBS was added to the Petri dish before transferring embryos evenly into Eppendorf tubes. For ORO extraction, 5–10 embryos were pooled per tube, PBS was removed, and 250 μL of 4% v/v ethanol in 100% isopropanol was added. Samples were briefly vortexed and incubated for 2 hours at room temperature on a plate shaker until zebrafish tissues were devoid of visible ORO staining, confirming complete extraction.

For quantification, 200 μL of the extracted ORO solution was transferred into individual wells of a 96-well plate. Optical density (OD) was measured at 495 nm following a 5-second plate shake. OD readings from ORO-stained embryos were normalized against those from non-stained embryos (blank).

### Establishing and monitoring the zebrafish xenograft model

A zebrafish xenograft model was developed to study the *in vivo* progression of TNBC cells and to evaluate both the efficacy and therapeutic index of EA extract.

#### Cell culture.

Human triple-negative breast cancer cells (MDA-MB-231) were cultured following American Type Culture Collection (ATCC; Rockville, MD, USA) guidelines. Cells were maintained in RPMI-1640 medium (Gibco, Life Technologies) supplemented with 10% fetal bovine serum (FBS; Life Technologies, CA, USA) and 1% penicillin/streptomycin (Sigma-Aldrich, MO, USA). Cultures were incubated at 37 °C in a humidified atmosphere containing 5% CO₂ and 95% O₂. Growth medium was replaced every alternate day to maintain optimal conditions for exponential cell proliferation, which was confirmed by microscopic observation before use in xenograft experiments.

#### Fluorescent labelling of MDA-MB-231 cells.

MDA-MB-231 breast cancer cells were routinely maintained in RPMI medium (Thermo Fisher Scientific) until the desired confluence was reached. For the fluorescent labelling step, the culture medium was replaced with Dulbecco’s Modified Eagle Medium (DMEM; Thermo Fisher Scientific), as recommended by the CM-DiI manufacturer to ensure optimal labelling efficiency and cell viability. Cells were prepared as a single-cell suspension at a density of 1 × 10⁶ cells/mL in DMEM, and 5 μL of CM-DiI Red Fluorescent Protein (RFP) dye (catalogue number V22888, Thermo Fisher Scientific) was added per mL of suspension and gently mixed to achieve uniform distribution. The suspension was incubated for 20 minutes at 37°C, after which the staining solution was removed, and the cells were washed three times with pre-warmed growth medium, with 10-minute intervals between washes, to eliminate unbound dye. Labelling efficiency was verified using an EVOS M5000 fluorescence microscope (Thermo Fisher Scientific) with an RFP filter. Cells were then harvested by trypsinization, pelleted at 1500 rpm for 5 minutes at 37°C, and resuspended in pre-warmed medium. This wash–centrifugation cycle was repeated twice to ensure complete removal of residual dye while preserving cell viability prior to xenotransplantation.

#### Preparation of zebrafish for microinjection.

Adult zebrafish were bred two days prior to microinjection. Fertilized eggs were collected the following morning and screened for viability, with healthy embryos maintained at 28 °C until two days post-fertilization (dpf). At this stage, coinciding withdechorionation, —embryos were prepared for microinjections.

Embryos were anesthetized in 0.03% tricaine methane sulfonate solution and positioned dorsally on an agarose-coated Petri dish to provide stability during injection. Microinjection needles were fabricated from borosilicate glass capillaries using a Sutter Instrument P-20 micropipette puller set to the following parameters: pull = 20, velocity = 50, time = 200, pressure = 200, and heat = ramp +21 °C, as described by Wehmas [59]. These parameters produced fine-tipped needles suitable for the precise delivery of cancer cells into the embryonic yolk sac.

#### Xenotransplantation of RFP labelled cells (MDA-MB-231) in zebrafish larvae.

The microinjection procedure served as the final step in introducing RFP-labelled human triple-negative breast cancer cells (MDA-MB-231) into zebrafish larvae, following the embryo preparation and cell-labelling steps described previously [60,61]. A borosilicate glass capillary needle was mounted onto the micromanipulator, and its tip was carefully trimmed with forceps to achieve optimal precision. The needle was then fitted with an Eppendorf capillary tip preloaded with 5 μL of the labelled cancer cell suspension, adjusted to deliver approximately 300 cells per larva. For accurate delivery, the microneedle was positioned at a 45° angle, and the cell suspension was injected directly into the yolk sac of each larva.

Experimental groups were established to evaluate the therapeutic effects of EA extract. The negative control group consisted of uninjected larvae maintained in E3 medium. The positive control group was injected with labelled cancer cells and maintained in E3 medium. Two treatment groups were injected with labelled cancer cells and subsequently exposed to EA at concentrations of 0.5 mg/mL or 0.75 mg/mL. Each group contained 30 embryos (n = 30), and all experiments were conducted in triplicate.

Following microinjection, larvae were transferred to 6-well culture plates for observation. While initial injection counts were recorded for each group, some larvae were excluded from subsequent analyses due to mortality, severe edema, or absence of detectable cancer cells at 1 day post-injection (dpi).

#### Imaging and monitoring of cancer cell proliferation.

Proliferation and metastatic potential of RFP-labelled MDA-MB-231 cells were assessed at 1 and 2 dpi using an EVOS M5000 fluorescence microscope (Thermo Fisher Scientific). At each time point, larvae from all groups were immobilized in methylcellulose and positioned on concave glass slides for imaging.

Fluorescence images were captured using an RFP filter under a light-sealed environment to minimize background noise. All imaging was conducted under identical conditions, including 4 × magnification, to ensure accurate cross-group comparisons. Image analysis quantified the presence, distribution, and intensity of RFP-labelled cancer cells, providing a basis for evaluating EA treatment efficacy on cancer cell proliferation and early dissemination.

#### Quantification of RFP-labelled cancer cells fluorescence.

To objectively assess the impact of EA on cancer cell burden and its potential effects on proliferative cell clusters and early dissemination within the xenograft model, we quantified the fluorescence intensity of RFP-labeled cancer cells from captured images. This quantification was performed using ImageJ software (version 1.54, National Institutes of Health, Bethesda, MD), which allowed for the measurement of the fluorescent signal intensity from the labeled cancer cells. Background fluorescence was subtracted from the raw measurements using ImageJ’s background subtraction tools, accurately representing the specific signal associated with the cancer cells. This background correction yielded the Corrected Total Cell Fluorescence (CTCF) value [62]. By comparing the CTCF values across all experimental groups (negative uninjected control, positive injected control, and injected with EA treatment groups), we gained valuable insights into the efficacy of the EA treatment. Higher CTCF values indicate a greater number of cancer cells or increased fluorescence intensity, potentially suggesting limited treatment effectiveness. Conversely, lower CTCF values may indicate a successful reduction in cancer cell burden or decreased fluorescence intensity, suggesting a positive treatment response through reduced cancer cell survival and proliferation.

#### Biodistribution assessment of RFP-labelled cancer cells.

Biodistribution and tumor progression analyses were performed to evaluate the spatial distribution and growth dynamics of RFP-labelled cancer cells within zebrafish larvae. Following microinjection, larvae from all experimental groups, including the negative uninjected control, positive injected control, and EA treatment groups were imaged using an EVOS M5000 fluorescence microscope (Thermo Fisher Scientific) at 24-hour intervals for up to 2 days post-injection (dpi).

Sequential imaging enabled comparative assessment of changes in both the size and localization of RFP-labelled cancer cell clusters across treatment groups. These observations provided insights into whether EA exposure influenced cancer cell dissemination and tumor formation within the zebrafish model. This analysis offered valuable evidence regarding EA’s potential to modulate cancer cell dissemination and its efficacy in targeting malignant cell behavior *in vivo*.

### Statistical analysis

All statistical analyses were performed using GraphPad Prism software (version 8; GraphPad LLC, Dotmatics). Differences between experimental groups were evaluated using one-way analysis of variance (ANOVA) followed by Šídák’s multiple comparison test. This approach allowed for the comparison of multiple treatment groups, namely, the positive control (20 µg/mL zinc oxide) and EA treatment groups—against the negative control (E3 medium, E3M). Each experimental group consisted of 30 embryos, and all experiments were conducted in triplicate. Statistical significance was set at *P* < 0.05. Levels of significance were indicated as follows*:* P < 0.05: *, P < 0.01: **, P < 0.001: ***, P < 0.0001: ****.

## Results

### Developmental toxicity of *Elaeagnus angustifolia* (EA) in zebrafish larvae

#### Survival analysis.

**Fig 1:**
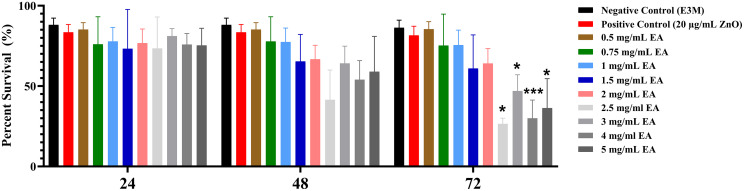
Survival rates of zebrafish larvae exposed to varying concentrations of Elaeagnus angustifolia (EA) at 24, 48, and 72 hours post-fertilization (hpf). Larvae were treated with EA at concentrations of 0.5, 0.75, 1.0, 2.0, 2.5, 3.0, 4.0, and 5.0 mg/mL, along with a positive control (20 µg/mL zinc oxide) and a negative control (E3 medium). Survival was assessed by manually counting live embryos under a standard microscope and expressing the result as a percentage of the initial number of embryos. Data are presented as mean ± standard deviation (% survival; n = 30 embryos per group, experiment performed in triplicate). Statistical analysis was performed using one-way ANOVA followed by Šídák’s multiple comparisons test. Significant differences relative to the negative control are indicated as follows: p < 0.05, **p < 0.001.

The impact of EA on zebrafish larval survival was assessed in a concentration-dependent manner. Larvae were exposed to a range of EA concentrations (0.5, 0.75, 1.0, 2.0, 2.5, 3.0, 4.0, and 5.0 mg/mL) and monitored at 24-hour intervals up to 72 hpf. As illustrated in Fig 1, statistically significant reductions in survival were observed only at the 72-hour time point. Higher EA concentrations (2.5–5.0 mg/mL) resulted in a marked decrease in survival compared with the negative control group (E3 medium), with the most pronounced effect recorded at 4.0 mg/mL (p < 0.001). In contrast, lower concentrations (≤ 2.0 mg/mL) did not significantly influence survival, demonstrating a clear concentration-dependent toxicity profile. The reference toxicant group (20 µg/mL ZnO nanoparticles), included to assess sublethal developmental and physiological effects rather than to induce mortality, exhibited no significant difference in survival compared with the negative control. This outcome was consistent with the intentional selection of a sublethal concentration based on prior evidence, allowing differentiation between mortality-driven and non-lethal endpoints in the zebrafish assay.

#### Hatching rate analysis.

The hatching rate, defined as the percentage of embryos that successfully emerged from their chorions, was evaluated at 72 hpf to identify potential developmental delays induced by EA exposure. The rate was calculated by dividing the number of hatched embryos by the total number of embryos in each group. Under normal conditions, zebrafish larvae begin hatching between 48 and 52 hpf, with the process typically completed by 72 hpf.

**Fig 2:**
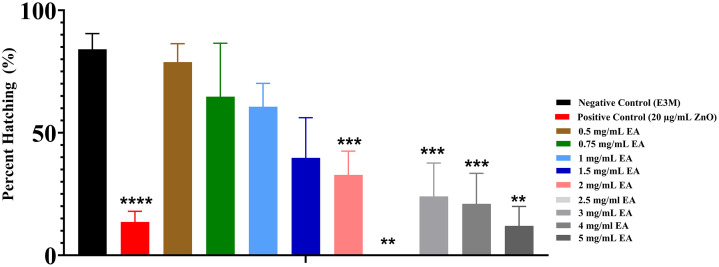
Hatching rate of zebrafish embryos at 72 hpf following exposure to varying concentrations of Elaeagnus angustifolia extract (EA). Hatching rate was determined as the proportion of embryos successfully emerging from their chorions after exposure to EA concentrations of 0.5, 0.75, 1.0, 1.5, 2.0, 2.5, 3.0, 4.0, and 5.0 mg/mL, alongside a negative control (E3M) and a positive control (20 µg/mL Zinc Oxide). Data are presented as mean ± standard deviation (% hatching) for n = 30 embryos per group, with experiments performed in triplicate. Statistical analysis was conducted using one-way ANOVA followed by Šídák’s multiple comparisons test. Significant differences relative to the negative control group (E3M) are indicated as follows: ****p < 0.0001.

As shown in Fig 2, EA concentrations ≥ 2.0 mg/mL resulted in a significant, dose-dependent reduction in hatching success compared to the negative control group (E3M). In contrast, lower concentrations (≤ 1.5 mg/mL) did not significantly affect hatching rates. Consistent with expectations, the positive control group (20 µg/mL ZnO) exhibited a markedly reduced hatching rate (p < 0.0001), confirming the assay’s sensitivity in detecting EA-induced developmental impairments in zebrafish embryos.

#### Cardiotoxicity assessment.

Following the hatching rate analysis, cardiac function was assessed in the dorsal aorta (DA) of zebrafish larvae at 72 hpf. Only EA concentrations ≤ 2 mg/mL were included, as higher concentrations had previously demonstrated adverse effects on both survival and hatching rates.

**Fig 3:**
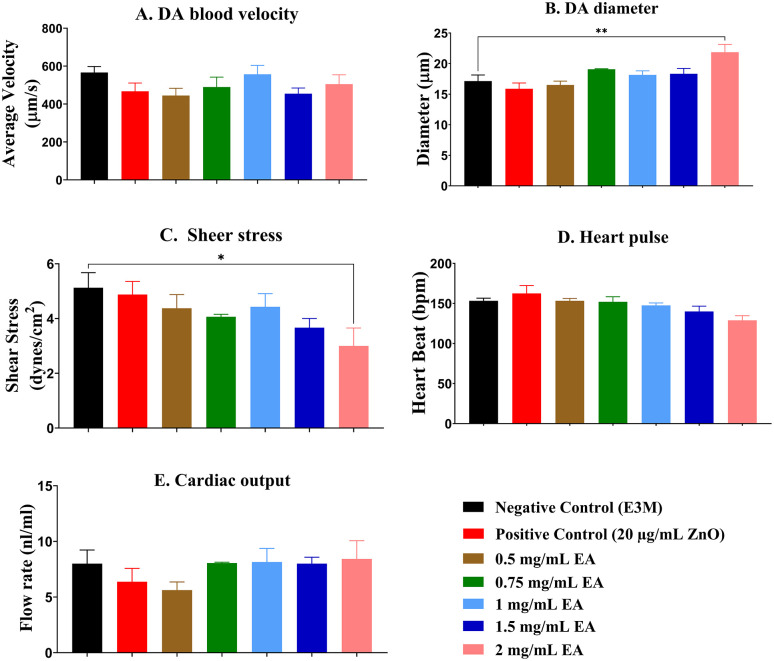
Cardiac parameter assessment in the dorsal aorta of zebrafish larvae at 72 hours post-fertilization (hpf). Zebrafish larvae were exposed to varying concentrations of Elaeagnus angustifolia (EA) extract (0.5, 0.75, 1.0, 1.5, 2.0 mg/mL), along with a positive control (20 µg/mL Zinc Oxide) and a negative control (E3M). Cardiac function was evaluated by measuring: (A) dorsal aortic blood flow velocity (µm/s), (B) vessel diameter (µm), (C) shear stress (dynes/cm²), (D) heart rate (beats per minute, bpm), and (E) flow rate (nL/min). Data are presented as mean ± standard error of the mean (SEM) (n = 5 per group). Statistical significance was determined using one-way ANOVA followed by Šídák’s multiple comparisons test. Significant differences relative to the negative control group (E3M) are indicated by asterisks: *p < 0.05, **p < 0.01.

As shown in Fig 3A, no significant differences in aortic blood flow velocity were detected across any of the tested concentrations. However, Fig 3B reveals a significant increase in vessel diameter at 2 mg/mL compared to the negative control group (E3M). Correspondingly, Fig 3C demonstrates a significant reduction in aortic shear stress at the same concentration (p < 0.05). Other cardiovascular parameters, including heart rate (Fig 3D) and flow rate (Fig 3E), remained unaffected by EA exposure and did not exhibit any consistent trends across the tested concentration range.

#### Neuromuscular toxicity: Behavioral and locomotion assay.

A behavioral locomotion assay was conducted at 96 hpf to evaluate the potential neurobehavioral effects of EA exposure on zebrafish larvae. Swimming activity was assessed by quantifying both the total and average distances traveled. Following a 20-minute acclimatization period, larvae were subjected to alternating 10-minute light and dark cycles over a 60-minute recording session.

**Fig 4:**
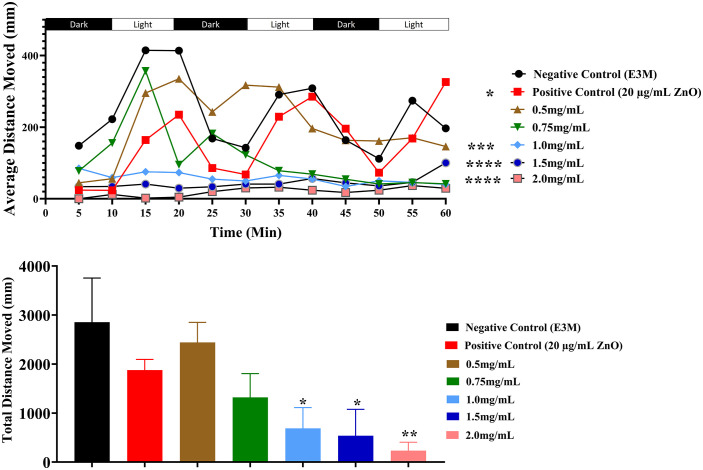
Behavioral and locomotion assay of zebrafish larvae at 96 hpf. Zebrafish larvae were exposed to varying concentrations of *Elaeagnus angustifolia* (EA) (0.5, 0.75, 1.0, 1.5, and 2.0 mg/mL), as well as to a positive control (20 µg/mL Zinc Oxide) and a negative control (E3M). At 96 hpf, individual larvae were transferred to 96-well plates placed within the Viewpoint ZebraLab chamber and subjected to alternating 10-minute light and dark cycles for a total of 60 minutes. Locomotor activity was recorded to quantify both **(A)** the average distance traveled every 5 minutes during the assay and **(B)** the total distance traveled at the end of the recording period. Data are expressed as mean ± standard error of the mean (SEM) (n = 10 larvae per group; experiments performed in duplicate). Statistical analysis was conducted using one-way ANOVA followed by Šídák’s multiple comparisons test. Significant differences relative to the negative control (E3M) are indicated as follows: *P < 0.05, **P < 0.01, ***P < 0.001, ****P < 0.0001.

EA exposure induced a clear, concentration-dependent reduction in locomotor activity. Compared with the negative control group (E3M), larvae exposed to 1.0 mg/mL EA exhibited a significant decrease in both average distances traveled (p < 0.001) and total distance traveled (p < 0.05) (Fig 4A and 4B). This effect was amplified at higher concentrations: 1.5 mg/mL EA significantly reduced the average distance (p < 0.05) and total distance (p < 0.01) traveled, while 2.0 mg/mL EA caused a marked decrease in both average (p < 0.0001) and total (p < 0.01) movement compared with the negative control. Larvae in the positive control group (20 µg/mL Zinc Oxide) also demonstrated a significantly lower average distance traveled, confirming the assay’s sensitivity in detecting neuromuscular impairment.

Although high EA concentrations substantially reduced swimming distances, the characteristic activity pattern—higher movement during light cycles and reduced activity in dark cycles—remained intact across all groups. This indicates that while baseline light–dark responsiveness was preserved, EA exposure impaired the magnitude of locomotor responses, suggesting potential neuromuscular toxicity and diminished swimming performance in treated larvae.

#### Hepatotoxicity: Oil red O staining.

**Fig 5:**
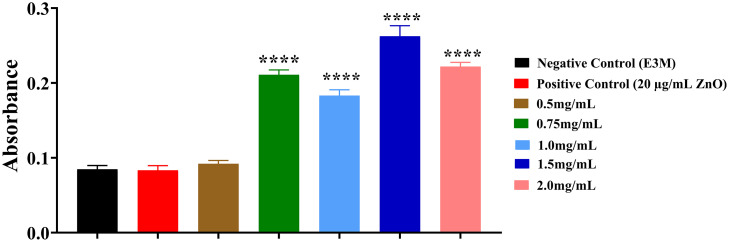
Oil Red O staining for lipid accumulation in zebrafish livers at 5 days post-fertilization (dpf). Larvae were exposed to varying concentrations of Elaeagnus angustifolia (EA) (0.5, 0.75, 1.0, 1.5, and 2.0 mg/mL), and Oil Red O staining intensity was measured to quantify neutral lipid deposition in the liver. Data are presented as mean ± standard error of the mean (SEM) (n = 3 embryos per group; experiment performed in duplicate). Statistical significance was determined using one-way ANOVA followed by Šídák’s multiple comparisons test. Significant differences from the negative control group (E3M) are indicated by ****p < 0.0001.

An Oil Red O (ORO) staining assay was performed at 5 days post-fertilization (dpf) to evaluate the potential hepatotoxic effects of EA exposure, focusing on hepatic lipid accumulation. This histological method enables both visualization and quantification of neutral lipids within liver tissue. As shown in Fig 5, larvae exposed to EA concentrations ≥0.75 mg/mL displayed a marked increase in ORO absorbance in the liver compared with the negative control group (E3M) (p < 0.0001). This was visually evident as a deeper red coloration in the hepatic region, indicative of elevated neutral lipid deposition. Increasing EA concentrations resulted in progressively more intense staining, demonstrating a clear dose-dependent effect. These results suggest that higher EA doses may impair lipid homeostasis and contribute to hepatic steatosis in zebrafish larvae.

During the dose optimization phase, higher concentrations of EA induced marked organ toxicity across all evaluated systems and were therefore excluded from subsequent xenograft experiments to ensure animal welfare. In contrast, concentrations of 0.5 mg/mL and 0.75 mg/mL produced minimal adverse effects in most organ toxicity assays and showed no evidence of teratogenicity in zebrafish embryos, as confirmed by comprehensive toxicity evaluations. The selection of 0.75 mg/mL for further work was based on its position at the threshold between no observable adverse effect and the onset of measurable developmental or physiological changes in our zebrafish model. While hepatotoxicity at 0.75 mg/mL was not significantly greater than at some higher concentrations, this dose represented the highest concentration that did not cause significant reductions in overall survival or severe morphological defects, yet still elicited detectable sublethal responses in hepatic endpoints, thereby enabling mechanistic exploration without confounding effects from high mortality. Furthermore, 0.75 mg/mL consistently produced mild but quantifiable changes across multiple parameters, including biochemical and histological markers, making it suitable for follow-up work aimed at understanding early toxicological pathways rather than overt lethality. Although doses between 0.5 and 0.75 mg/mL might have provided finer resolution, the initial screening was designed with broader concentration spacing to map the overall toxicity profile, allowing both sublethal and lethal ranges to be identified before narrowing in on the threshold dose. Accordingly, 0.5 mg/mL and 0.75 mg/mL were selected for the xenograft experiments to investigate the potential anticancer activity of EA while minimizing harm to the model organism.

#### Establishing a zebrafish xenograft model.

**Fig 6:**
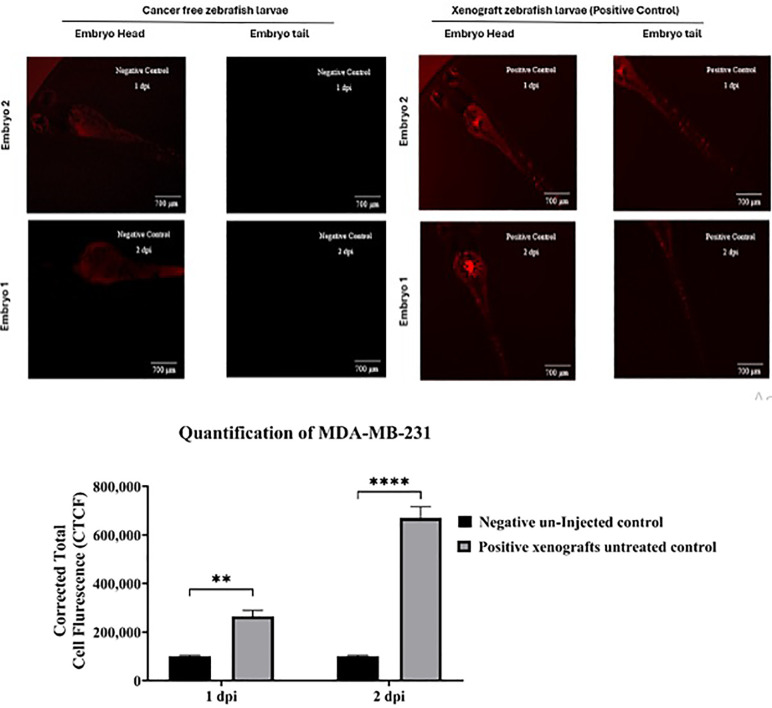
Fluorescence imaging of cancer-free and xenograft zebrafish larvae injected with triple-negative breast cancer (TNBC) cells (MDA-MB-231). **(A)** At 48 hpf, zebrafish larvae in the xenograft group were injected with red fluorescent protein (RFP)-labeled MDA-MB-231 cells, while uninjected larvae served as negative controls. Images were acquired at 4 × magnification to visualize RFP fluorescence in the head and tail regions at 1 and 2 days post-injection (dpi). The left panels depict cancer-free larvae (negative control), while the right panels show xenografted larvae (positive control). For each group, representative images are presented for two individual embryos (Embryo 1 and Embryo 2), with the head region shown in the left column and the tail region in the right column. Scale bars = 700 µm. (**B)** Data are presented as mean ± standard error of the mean (SEM) (n = 6 embryos per group; experiment performed in duplicate). Statistical significance was determined using two-way ANOVA followed by Šídák’s multiple comparisons test, with differences relative to the untreated xenograft control group indicated as **p < 0.01 and ****p < 0.0001.

A zebrafish xenograft model was developed to evaluate the anticancer potential of EA by xenotransplanting triple-negative breast cancer cells (MDA-MB-231) into zebrafish larvae at 48 hpf. Xenografted larvae were imaged at one and two days post-injection (dpi) and compared with negative, uninjected control embryos (Fig 6). Visual confirmation of successful engraftment was achieved by distinguishing the intrinsic autofluorescence of zebrafish embryos from the red fluorescent protein (RFP) signal emitted by the transplanted cancer cells.

By 1 dpi, TNBC cells were localized primarily at the yolk sac injection site, forming distinct aggregates that represented the primary tumor mass. Evidence of metastatic spread was also observed, with cancer cells migrating to other regions of the larvae, particularly towards the tail (Fig 6A). At 2 dpi, the number of cancer cells had significantly increased, indicating ongoing proliferation and expansion within the host (Fig 6B).

**Fig 7:**
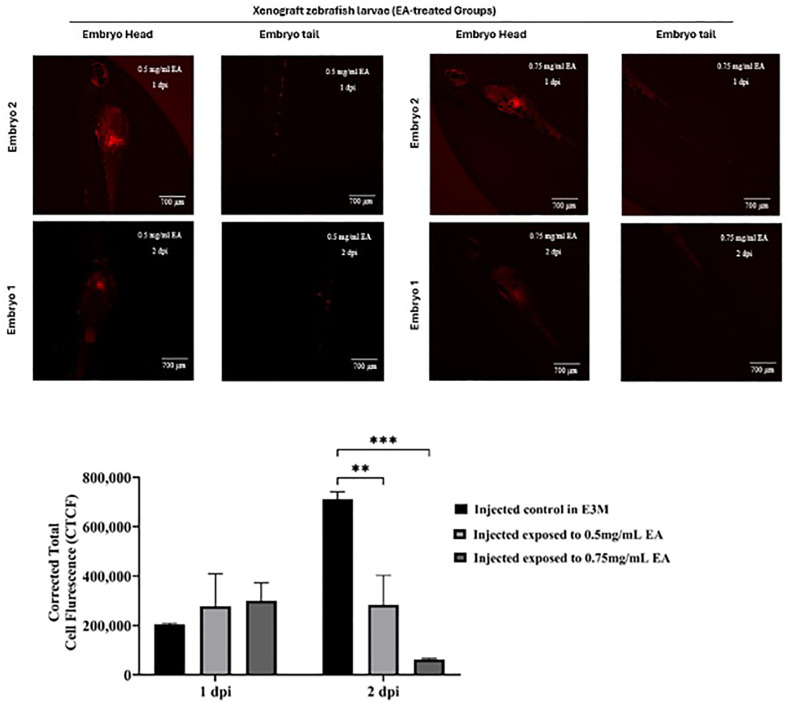
Zebrafish xenograft model injected with triple-negative breast cancer (TNBC; MDA-MB-231) cells at 48 hours post-fertilization (hpf). Images show xenografted larvae containing red fluorescent protein (RFP)-labeled TNBC cells at 1 and 2 days post-injection (dpi) at different concentrations (0.50 mg/ml and 0.75 mg/ml EA). **(A)** Representative fluorescence images of head and tail regions from xenografted larvae treated with 0.5 mg/mL EA (top) or 0.75 mg/mL EA (bottom) at both time points. **(B)** Quantitative analysis of RFP fluorescence intensity, reflecting tumor burden, in EA-treated xenografts compared to untreated xenograft controls. Data are expressed as mean ± standard error of the mean (SEM) (n = 6 embryos per group; experiment performed in duplicate). Statistical significance was determined by two-way ANOVA with Šídák’s multiple comparisons test, with differences compared to untreated xenograft controls denoted as **p < 0.01 and ***p < 0.001.

Quantitative fluorescence analysis supported these observations. At 1 dpi, xenografted larvae exhibited detectable RFP fluorescence, confirming stable engraftment of TNBC cells (Fig 7B). By 2 dpi, fluorescence intensity had increased significantly compared with uninjected controls (****p < 0.0001), reflecting robust cancer cell proliferation within the zebrafish model. This zebrafish xenograft platform provides a reliable and physiologically relevant system for assessing the anticancer efficacy of EA *in vivo*.

#### Xenograft zebrafish model exposed to EA.

To investigate the anti-cancer potential of EA while minimizing adverse effects on zebrafish, we employed a xenograft model in which zebrafish larvae were injected with triple-negative breast cancer (TNBC; MDA-MB-231) cells. Following microinjection, larvae were exposed to two non-toxic concentrations of EA (0.5 mg/mL and 0.75 mg/mL), as determined in prior toxicity assays, while control xenografts received E3 medium only. An additional group of negative uninjected control was included for comparison.

EA exposure markedly reduced the fluorescence intensity and burden of injected TNBC cells in a dose-dependent manner, with both concentrations showing significant reductions compared to untreated xenografts at both 1 and 2 days post-injection (dpi). The higher concentration (0.75 mg/mL) produced the greatest effect, with a 42.6% reduction in mean tumor area at 2 dpi relative to untreated xenografts, while 0.5 mg/mL produced a 27.8% reduction (Fig 7A). Furthermore, EA treatment diminished the characteristic metastasis of TNBC cells to the tail region, with 0.75 mg/mL EA reducing metastatic fluorescence signal in the tail by 48.3% at 2 dpi, suggesting a potential correlation between treatment duration and metastasis suppression (Fig 7A).

Quantitative analysis of Corrected Total Cell Fluorescence (CTCF) supported these observations. Untreated xenografts exhibited a 1.9-fold increase in CTCF from 1 to 2 dpi, consistent with active cancer cell proliferation. In contrast, EA-treated xenografts displayed a dose-dependent suppression of fluorescence intensity at 2 dpi, with CTCF reduced by 31.5% at 0.5 mg/mL (p < 0.01) and 54.7% at 0.75 mg/mL (p < 0.001) compared with untreated xenografts (Fig 7B). Visual inspection of Figs 7A and 7B corroborated these findings, with EA-treated larvae demonstrating markedly lower tumor burden and reduced proliferation, whereas untreated xenografts showed intense fluorescence accumulation by 2 dpi.

Collectively, these quantitative and qualitative results provide compelling evidence that EA inhibits tumor growth and metastatic spread in the zebrafish xenograft model, supporting further investigation into its molecular mechanisms of action.

## Discussion

### Elaeagnus angustifolia (EA) as a potential anti-cancer agent for triple-negative breast cancer

EA, long recognized in traditional medicine for its antimicrobial, anti-inflammatory, and antioxidant properties [35], has gained attention for its potential anti-cancer activity. This study assessed the effects of EA against TNBC using a zebrafish xenograft model inoculated with the aggressive MDA-MB-231 cell line. TNBC presents a formidable therapeutic challenge due to its high metastatic capacity, rapid disease progression, and absence of hormone or HER2 targets. Although advances such as immune checkpoint inhibitors and PARP inhibitors have shown promise, their applicability remains limited to specific patient subsets and is often associated with substantial costs and side effects [63].

### The zebrafish xenograft model: Strengths and validation

A key strength of this work lies in employing a zebrafish xenograft model, which offers a physiologically relevant microenvironment not captured by in vitro assays [64,43]. Zebrafish embryos provide several advantages for cancer research, including rapid development, genetic similarity to humans, and optical transparency that enables real-time, in vivo imaging [65–67]

Our model was validated through the successful engraftment and proliferation of MDA-MB-231 cells, which adhered to the yolk sac and disseminated to distal regions, particularly the tail—a metastatic pattern consistent with previous zebrafish studies [27,34,68–72].. The observed intravasation and migration behaviors align with prior reports highlighting the high metastatic potential of this cell line, reinforcing the zebrafish xenograft as a robust system for evaluating tumor biology and therapeutic efficacy.

### Toxicity profiling and safe concentration determination

Prior to efficacy testing, we conducted comprehensive toxicity assessments to define a non-lethal concentration range for EA. Survival and hatching assays revealed a concentration-dependent impact on embryonic development and on critical cellular processes [73], with survival rates decreasing at ≥2.0 mg/mL and hatching rates affected from ≥1.0 mg/mL. Notably, the low hatching rate at 2.5 mg/mL, despite moderate survival (~65%), likely reflects delayed hatching rather than lethality consistent with non-monotonic dose–response patterns in zebrafish toxicology.

Additional endpoints provided further insight; a reduction in aortic shear stress and increased vessel diameter at 2.0 mg/mL suggested potential vascular effects [74,75]; significant reductions in larval locomotion at ≥1.0 mg/mL indicated possible neurotoxicity [76–78]; and elevated Oil Red O staining at ≥0.75 mg/mL suggested hepatic lipid accumulation and possible hepatotoxicity [79–82]

From these results, we identified 0.5–0.75 mg/mL as a safe exposure range. The 0.75 mg/mL dose was selected for further study as it represented the threshold between no observable adverse effects and the onset of mild, quantifiable, non-lethal physiological changes, providing an optimal balance between safety and biological activity. This approach ensures that any observed anti-cancer effects are attributable to targeted activity against tumor cells rather than generalized host toxicity.

### Anti-cancer efficacy

Within the established safe range, EA markedly suppressed tumor growth and metastatic dissemination in a dose-dependent manner. Corrected total cell fluorescence (CTCF) measurements confirmed reduced tumor burden, with 0.75 mg/mL producing the most pronounced inhibition of both primary tumor expansion and metastatic spread. These findings are particularly significant given the aggressive, drug-resistant nature of MDA-MB-231 cells.

Our results align with prior in vitro evidence showing that EA and its bioactive constituents, particularly flavonoids—can induce apoptosis via p53 activation, trigger cell cycle arrest, and suppress epithelial–mesenchymal transition [42,43,83–88]. Such multi-targeted actions are advantageous for TNBC, which lacks a single dominant therapeutic target. Furthermore, recent research has explored innovative delivery methods for EA, such as EA-loaded zinc oxide nanoparticles, which may enhance bioavailability and provide targeted delivery to cancer cells [89].

By incorporating molecular assays into the zebrafish platform, future work could bridge the gap between our current phenotypic observations and the underlying pathways of EA’s anti-cancer activity. Such integrative studies would not only strengthen the translational relevance of the zebrafish model but also help identify biomarkers for therapeutic monitoring and optimization of EA-based interventions.

### Mechanistic insights and future investigations

Although our zebrafish experiments did not directly examine molecular pathways, previous in vitro studies suggest several mechanisms that merit in vivo investigation. EA has been shown to activate caspase-3 and caspase-9, upregulate p53, and promote mitochondrial-mediated cell death, indicating a potential role in apoptosis induction that could be validated in zebrafish using apoptosis-specific reporters and Annexin V staining [42,43,90,91]. Phytochemicals one componanet in EA has been reported to induce G2/M phase cell cycle arrest through the modulation of cyclins B1 and D1 and cyclin-dependent kinases, along with increased expression of p21 and p27; these effects could be confirmed in vivo through live imaging with cell cycle–specific biosensors [43,92]. Furthermore, EA’s ability to upregulate E-cadherin while downregulating vimentin and fascin points to a possible inhibition of epithelial–mesenchymal transition, which could be investigated in zebrafish by tracking labeled tumor cell dissemination and assessing epithelial-mesenchymal transition marker expression [43]. Incorporating these molecular endpoints into zebrafish xenografts would not only clarify the pathways underlying EA’s anti-cancer effects but also strengthen the translational relevance of the model and help identify biomarkers for therapeutic monitoring.

Recent work by Imraish et al. (2024) demonstrated that EA-loaded zinc oxide nanoparticles possess anti-inflammatory and antioxidant properties and may enhance targeted delivery to cancer cells [89]. Combining safe-dose EA treatment with nanoparticle carriers in zebrafish could improve bioavailability and tumor-specific accumulation while minimizing off-target effects, warranting further preclinical exploration.

### Limitations and future directions

This study has several limitations that should be acknowledged. The inherent differences between zebrafish and mammalian tumor biology, particularly in immune system function, tumor microenvironment, and pharmacokinetics [93] limit the direct translatability of our findings. In addition, the two-day observation period restricted our ability to evaluate long-term outcomes, including recurrence and delayed toxicities. Moreover, because zebrafish xenografts do not form mature tumors within 24–48 hours, our findings reflect EA’s effects on the proliferation and dissemination of injected TNBC cells rather than on established tumor masses. A limitation of this study is that the observed anti‑tumor effects of EA in zebrafish xenografts are primarily phenotypic. Future work will focus on dissecting the underlying molecular pathways, including EMT, p53, and angiogenesis, to provide mechanistic insight into EA’s activity against TNBC. Another limitation is the absence of direct comparisons with established TNBC therapies, such as PARP inhibitors, immune checkpoint inhibitors, or antibody–drug conjugates. PARP inhibitors (e.g., olaparib, talazoparib) have demonstrated clinical benefit in patients with germline BRCA mutations [94], but their efficacy remains limited to a subset of TNBC cases and resistance often develops. Immune checkpoint inhibitors (e.g., pembrolizumab, atezolizumab) represent another important therapeutic avenue [95], particularly in PD-L1–positive TNBC [96], yet their use is constrained by immune-related toxicities and variable patient response rates. Antibody–drug conjugates (e.g., sacituzumab govitecan) have recently shown promise by selectively delivering cytotoxic payloads to TNBC cells [97], but issues such as cost, accessibility, and adverse event profiles remain challenges. Comparing EA directly with these agents, or testing it in rational combination regimens, could provide valuable insights into its relative efficacy, potential synergistic effects, and therapeutic positioning within the TNBC treatment landscape.

Future studies should therefore extend the observation period in zebrafish models to evaluate long-term efficacy, recurrence, and late toxicities, while also incorporating head-to-head comparisons with clinically approved TNBC therapies. Rational combination studies may reveal whether EA enhances the activity of existing agents or mitigates their toxicity, thereby offering a complementary therapeutic strategy. Furthermore, molecular analyses including gene expression profiling, immunofluorescence, and live imaging will be essential to validate in vitro mechanistic hypotheses in vivo, clarifying whether pathways such as apoptosis induction, cell cycle arrest, and epithelial-mesenchymal transition inhibition underlie the observed anticancer effects. Ultimately, progression to mammalian models is indispensable to confirm safety, establish pharmacokinetic and pharmacodynamic parameters, and strengthen the clinical translatability of EA as a potential therapeutic candidate for aggressive breast cancer subtypes.

## Conclusion

This work provides preliminary in vivo evidence that EA extract can inhibit tumor growth and metastasis of triple-negative breast cancer in a zebrafish xenograft model at non-toxic concentrations. The observed dose-dependent efficacy, combined with a favorable safety profile, underscores EA’s potential as a low-toxicity, multi-targeted therapeutic candidate. These findings lay the groundwork for mechanistic studies, advanced delivery strategies, and validation in mammalian systems to support EA’s potential integration into TNBC management.

## Supporting information

S1 File Copy of Row data and statistical analysis. (XLSX)
